# Supplementary material for: Feedback for Emergency Ambulance Staff: A National Review of Current Practice Informed by Realist Evaluation Methodology
Source: Healthcare (Basel). 2023 Aug 8;11(16):2229. doi: 10.3390/healthcare11162229 (PMC10454701; doi:10.3390/healthcare11162229)
Supplement: Supplementary file 1 [file healthcare-11-02229-s001.zip › Supplement File S1. SurveyMeasure.pdf]

Supplement File S1. Survey measure

Collecting Information about Person Completing the Survey

- Name of person completing the survey
- Email address of person completing the survey
- Role of person completing the survey
- Role in relation to the prehospital feedback initiative: Programme lead, team member

Details about the Prehospital Feedback Initiative

- Name of initiative
- Select which NHS ambulance trust this prehospital feedback initiative is linked with: [*insert list of NHS ambulance trusts, multiple selection possible*]
- Please state which hospital trust this prehospital feedback initiative is linked with
- Is this initiative currently active?
- When was this initiative first implemented?
- Who developed this initiative?
- How many feedback reports has this prehospital feedback initiative provided since implementation? (selection of <10, 10-50, 50-100, 100-200, 200-300, 300-400, 400-500, >500)

Mechanisms of the Prehospital Feedback Initiative

- Recipient of feedback: Frontline ambulance staff/Helicopter emergency medical staff/Ambulance service managers/Emergency Operations Centre staff/Ambulance service as an organisation
- Source of feedback
- Content of feedback
- Mode of feedback
- Format of feedback (including visual or graphical elements)
- Frequency of feedback
- Lag-time of feedback being provided
- Time/resources involved in generating feedback
- Recipient level: individual/team/organisation
- Patient cases: individual/aggregate
- Do recipients request feedback (pull model) or is it provided without seeking (push model): pull/push/both
- Was this initiative implemented as a stand-alone project or as part of a multifaceted intervention: stand-alone/multi-faceted
- Instructions for improvement/action plan included?: Yes/No
- Underlying theory: Yes______________/No

Implementation of the Prehospital Feedback Initiative

- What were the barriers to implementation?
- What were the facilitators?

Consequences of the Prehospital Feedback Initiative

- What were the specific aims of this prehospital feedback initiative?
- Did the project achieve what you hoped it would?

Closing Questions and Statement

- What do you think are the most important characteristics of this prehospital feedback initiative?
- What makes this initiative different from other prehospital feedback initiatives?
