# Supplementary material for: Feedback for Emergency Ambulance Staff: A National Review of Current Practice Informed by Realist Evaluation Methodology
Source: Healthcare (Basel). 2023 Aug 8;11(16):2229. doi: 10.3390/healthcare11162229 (PMC10454701; doi:10.3390/healthcare11162229)
Supplement: Supplementary file 1 [file healthcare-11-02229-s001.zip › Supplement File S2. InterviewGuideLeads.pdf]

Supplement File S2. Interview guide for feedback initiative leads

Section A: Background information

1. Please tell me about your current role and provide a brief outline of what this involves
2. How long have you been in this role?
3. How many years have you been working at this ambulance service/hospital trust [*delete as appropriate*]?
4. Could you talk me through your involvement with this prehospital feedback initiative?
5. What is the prehospital feedback initiative called locally?
6. Can you tell us a bit more about the setting where this prehospital feedback initiative is located? [*For emergency department-based initiatives: type of emergency department, geographical area, staff skill mix, patient mix. For ambulance service-based initiatives: local call volume, geographical area, staff skill mix.*]

Section B: Initiative context and motivation

7. Thinking back, how did you first become involved in prehospital feedback?
8. When did you first decide to do something locally regarding prehospital feedback?
    a. How did this initiative come about?
    b. Was there a specific problem you were trying to address?
9. What were the original aims and motivations for starting the project?
    a. Why was that important? Why was this on the agenda at the time?
    b. What were you trying to change with the project?

Section C: The design and mechanisms of the prehospital feedback initiative

10. Let's talk about the development of the initiative. How was it actually designed?
    a. Who was involved? Staff/users, developers?
    b. What were the key design decisions?
    c. What was the rationale/theory underpinning these decisions?
11. I'd like to find out a little more specifically about how you think your initiative should ideally work. Can you explain this to me in as much detail as possible? [*Confirm any items relating to this initiative which were unclear from online data collection instrument completed in Work stream 2*]
12. What are the resources involved in this initiative? What are the costs?

Section D: Initiation and implementation of the prehospital feedback initiative

13. Now, I want to talk about how it actually went when you first implemented the initiative.
    a. Could you give us an idea of the project timeline and key project dates?
14. How was the initiative set up?
    a. How many staff were involved directly in the project?
    b. Funding, equipment, technical support?
15. How was the initiative introduced to staff?
    a. Any training? Incentives?
16. What were your initial intentions?
    a. Did you implement the initiative more widely after success in one location?

Section E: Evaluation of the initiative

17. This last section is about evaluation of your initiative. So, taken as a whole, how would you evaluate the initiative?
    a. Did the initiative achieve what you hoped it would?
    b. What other effects did the initiative have? Did that come as a surprise for you?
18. How would you evaluate the implementation of the initiative when it first started?
    a. Were there any issues, what were these, how were they addressed?
    b. What were the main barriers to the design and implementation of your initiative? [*prompt to cover patient confidentiality, time & effort involved in generating feedback*]
    c. Did you adjust your initiative when you faced barriers? If yes, how?
19. How did ambulance staff react to your initiative initially?
    a. Did that change over time?
    b. Were there any concerns about the initiative expressed by staff?
20. How did hospital staff reach to your initiative initially?
    a. Did that change over time?
    b. Were there any concerns about the initiative expressed by staff?
21. How well was the initiative adopted by ambulance staff? [*confirm number of staff using the initiative as provided in data collection instrument of Work stream 2*]
22. Overall, what do you think has been the impact of this initiative on clinical practice?
    a. What effect do you think this prehospital feedback initiative has on individual prehospital clinicians?
    b. What about at a team-level? Organisational-level?
23. Have you had any feedback from senior leadership or stakeholders regarding this initiative?
24. Will the project be sustainable?
    a. If yes, how has this been achieved? If no, how could this be achieved?

    b. What are the plans for this initiative going forwards?

25. What do you think are the most important characteristics of this prehospital feedback initiative?
    c. What makes this initiative different from other prehospital feedback initiatives?
    d. What would you do differently if you were planning an initiative like this again?
26. Is there anything else you would like to say before we end our time together?
