# Supplementary material for: Feedback for Emergency Ambulance Staff: A National Review of Current Practice Informed by Realist Evaluation Methodology
Source: Healthcare (Basel). 2023 Aug 8;11(16):2229. doi: 10.3390/healthcare11162229 (PMC10454701; doi:10.3390/healthcare11162229)
Supplement: Supplementary file 1 [file healthcare-11-02229-s001.zip › Supplement File S3. InterviewGuideRecipients.pdf]

Supplement File S3. Interview guide for feedback recipients

Section A: Background information

1. Please tell me about your current role and provide a brief outline of what this involves
2. How long have you been in this role?
3. How many years have you been working at this ambulance service/hospital trust [*delete as appropriate*]?
4. Could you talk me through your involvement with this prehospital feedback initiative?
5. What is the prehospital feedback initiative called locally?
6. Can you tell us a bit more about the setting where this prehospital feedback initiative is located? For emergency department-based initiatives: type of emergency department, geographical area, staff skill mix, patient mix. For ambulance service-based initiatives: local call volume, geographical area, staff skill mix.

Section B: Perception of initiative rationale

7. When did you first become aware of the initiative?
    a. How were the aims and rationale communicated to you?
8. What is your understanding of the initiative aims and how it works?
    a. When the initiative was first introduced did you think it would be useful?
        - For you? For others? For the team? For the organisation?
    b. What was it like working here at the time the prehospital feedback initiative was introduced?
9. What did you hope to get out of engaging with the initiative?

Section C: Experience of using the prehospital feedback initiative

10. How do you use the prehospital feedback initiative?
    a. Did you feel like you understood how to use the initiative?
    b. Was it easy to use it? Did it fit in with your usual work activities?
        - How long did it take? How much effort was involved?
    c. When and how have you used the prehospital feedback initiative? Can you give us an example?
        - What motivated you to request feedback in these specific examples?

- What did you hope to achieve and why?

11. What guidance did you receive on using the initiative?
12. How did you feel about the initiative when it was first introduced?
    a. What motivated you to engage with the initiative?
    b. Did you have any concerns about the initiative?

Section D: Evaluation of impact on practice

13. Has the initiative impacted on your own professional practice?
    a. If so, how and in what ways?
    b. What did you get out of the project?
14. Do you think there was an effect was on the broader team/organisation?
    a. If so, how and in what ways?
    b. Any impact on working relationships? Teamwork?
15. Do you think the initiative has impacted on staff wellbeing? If yes, how?
16. Do you think the initiative has impacted on patient safety? If yes, how?
17. Are there any other ways for you to receive feedback within your role?
    a. If yes, what are they and how do they differ from this prehospital feedback initiative?
    b. If no, what do you think the consequences are of not receiving feedback? Specifically, to the quality and safety of patient care?
18. Do you plan to engage with this prehospital feedback initiative again?
19. What were the limitations of the initiative in your view?
20. What do you think will be the long-term benefits of the project?
21. From your point of view, how could the project be improved?
22. Is there anything else you'd like to say before we end our time together?
